# Ablation of beta subunit of protein kinase CK2 in mouse oocytes causes follicle atresia and premature ovarian failure

**DOI:** 10.1038/s41419-018-0505-1

**Published:** 2018-05-03

**Authors:** Qiu-Xia Liang, Zhen-Bo Wang, Fei Lin, Chun-Hui Zhang, Hong-Mei Sun, Liang Zhou, Qian Zhou, Heide Schatten, Filhol-Cochet Odile, Boldyreff Brigitte, Qing-Yuan Sun, Wei-Ping Qian

**Affiliations:** 1 Department of Reproductive Medicine, Peking University Shenzhen Hospital, 518036 Shenzhen, Guangdong China; 2 State Key Laboratory of Stem Cell and Reproductive Biology, Institute of Zoology, Chinese Academy of Sciences, 100101 Beijing, China; 3 University of Chinese Academy of Sciences, 100101 Beijing, China; 4 Center for Reproductive Medicine, Nanjing Drum Tower Hospital, The Affiliated Hospital of Nanjing University Medical School, 210008 Nanjing, China; 5 Department of Veterinary Pathobiology, University of Missouri, Columbia, MO 65211 USA; 6 INSERM U1036, Institute de Recherches en Technologies et Sciences pour le Vivant/Biologie du Cancer et de l’Infection, Commissariat à l’Énergie Atomique et aux Énerigies Alternatives Grenoble, Grenoble, France; 7 KinaseDetect, Krusaa, Denmark

## Abstract

Premature ovarian failure (POF), a major cause of female infertility, is a complex disorder, but the molecular mechanisms underlying the disorder are only poorly understood. Here we report that protein kinase CK2 contributes to maintaining follicular survival through PI3K/AKT pathway and DNA damage response pathway. Targeted deletion of CK2β in mouse oocytes from the primordial follicle stage resulted in female infertility, which was attributed to POF incurring by massive follicle atresia. Downregulated PI3K/AKT signaling was found after CK2β deletion, indicated by reduced level of phosphorylated AKT (S473, T308, and S129) and altered AKT targets related to cell survival. Further studies discovered that CK2β-deficient oocytes showed enhanced γH2AX signals, indicative of accumulative unrepaired DSBs, which activated CHK2-dependant p53 and p63 signaling. The suppressed PI3K/AKT signaling and failed DNA damage response signaling probably contribute to large-scale oocyte loss and eventually POF. Our findings provide important new clues for elucidating the mechanisms underlying follicle atresia and POF.

## Introduction

Premature ovarian failure (POF) refers to amenorrhea in women of less than 40 years of age accompanied by elevated menopausal levels of serum gonadotropins (follicle-stimulating hormone, FSH > 40 IU/l) and decreased estrogen^1^. POF is an ovarian dysfunction characterized by premature depletion of ovarian follicles in ~1% of women under the age of 40 years and 0.1% under the age of 30 years^2^, which usually leads to female infertility. The causes of POF vary and are complex, and it includes genetic aberrations^3–5^, autoimmune ovarian damage^6,7^, therapeutic interventions such as radiotherapy^8^ and chemotherapy^9^, with genetic factors being the main causes. Accumulating genes in the X chromosome such as FMR1^4,10^, FMR2^11^, BMP15^12–14^ and genes in the autosome such as FOXL2^15,16^, FSHR^17^, LH receptor^18^, and inhibin A^19,20^ are known to be involved in POF; however, for many years, the underlying mechanisms of POF have largely remained unknown.

Protein kinase CK2 is a ubiquitous serine/threonine protein kinase that is a heterotetramer consisting of two regulatory subunits, CK2β, and two catalytic subunits, CK2α and CK2α’^21^. Depending on specific functions, the catalytic and regulatory subunits may exist in the forms of α2β2, α’2β2, αα’β2, or unassembled molecules^22,23^. CK2 displays functions in numerous cellular processes by participating in multiple signaling pathways, including PI3K/AKT^24–27^, Wnt/β-catenin^28–31^, and the DNA damage response pathway^32–36^. Extensive studies have shown that CK2 is involved in the promotion of cell survival and anti-apoptotic functions in normal and cancer cells. In HEK-293T cells, CK2 phosphorylates AKT/PKB at Ser129, preventing AKT Thr308 dephosphorylation and enhancing the catalytic activity of AKT/PKB^24,30,37^, which in turn phosphorylates β-catenin at Ser552 and promotes its nuclear localization, eventually strengthening resistance to apoptosis by upregulating anti-apoptotic survivin gene transcription^30^. CK2 regulates PI3K signaling by phosphorylating PTEN C terminus^38,39^, which enhances the stability and inhibits the activity of PTEN^38–40^. In leukemia cells, CK2 inhibits Ikaros, a tumor suppressor, which can repress the transcription of genes promoting the PI3K pathway^27^. Since CK2 was first reported to facilitate DNA single-strand breaks repair^33^, accumulating evidence has shown that CK2 is involved in DNA damage repair pathway. CK2 regulates the G1/S DNA damage checkpoint by targeting p53^41,42^, p53 regulatory proteins (e.g., MDM-2)^43,44^ and Cdk-inhibitory proteins such as p21^WAF1/CIP1^ ^45,46^ and p27^KIP1^ ^47^. CK2 also contributes to the G2/M damage checkpoint by targeting or interacting with proteins such as CHK1^22^, CHK2^48^ and BRCA1^49^. Generally, CK2 functions as a monitoring hub of the cell cycle and apoptosis, integrating diverse signals into the appropriate cellular responses.

*CK2α*^*−/−*^ embryos die in mid-gestation, with defects in heart and neural tube^50^. *CK2α’*^*−/−*^ females show normal fertility, but males are infertile. Male mice lacking CK2α’ show extensive germ cell apoptosis characterized by nuclear abnormalities ranging from spermatogonia to early spermatids^51,52^. *CK2β*^*−/−*^ mice die shortly after implantation with no signs of apoptosis but reduced cell proliferation. *CK2β*^*−/−*^ blastocysts cannot develop an inner cell mass in vitro^53^. Zygote-specific knockout of *CK2β* is destructive for embryonic stem cells and primary embryonic fibroblasts^53^. The above studies demonstrate that CK2β is indispensable for cell survival. However, the roles of CK2β in folliculogenesis/oogenesis are largely unknown. Here we targeted CK2β for deletion in oocytes from the primordial follicle stage by crossing *Ck2β*^*fl/fl*^ mice with *Gdf9*-*Cre* mice. We found that CK2β was essential for female fertility and loss of CK2β caused ovarian follicle atresia and POF.

## Results

### Oocyte-specific deletion of *Ck2β* gene causes mice infertility

**Fig. 1:**
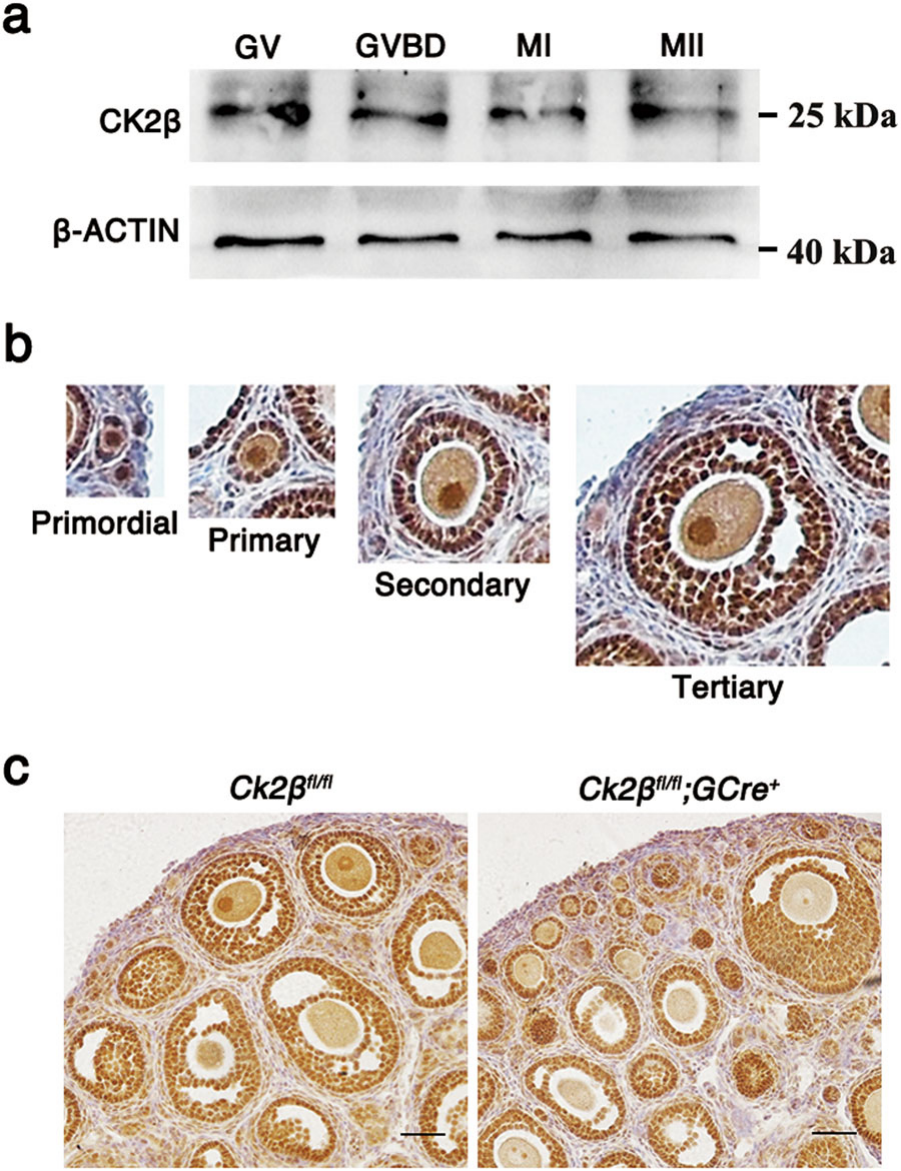
The expression and oocyte-specific deletion of CK2β. **a** Immunoblotting showing the expression pattern of CK2β in mouse oocytes at different developmental stages. A total 150 oocytes were collected after being cultured for 0, 4, 8, and 12 h, corresponding to GV, GVBD, MI, and MII stages, respectively. β-actin was detected as an internal control. **b** Immunohistochemistry analysis of the expression pattern of CK2β in primordial, primary, secondary, and tertiary follicles at PD21. **c** Immunohistochemistry detection of CK2β loss in oocytes of *Ck2β*^*fl/fl*^*;GCre*^*+*^ mice. Scale bar: 50 μm

Toward determining the potential roles of CK2β in the female reproductive system, expression patterns of CK2β in ovaries and oocytes at different developmental stages were assessed through immunoblotting and immunohistochemistry. Immunoblotting revealed that CK2β was stably and highly expressed in GV, GVBD, MI, and MII oocytes (Fig. 1a). Within the ovary, immunohistochemistry revealed that CK2β located in the nuclei of oocytes and granulosa cells from primordial follicles to antral follicles (Fig. 1b). These data suggest that CK2β potentially functions in folliculogenesis/oogenesis.

To confirm our hypothesis, we generated oocyte-specific *CK2β* mutant mice by crossing *Ck2β*^*fl*^ mice in which exon I–II were targeted with transgenic mice expressing *Gdf9* promotor-driven Cre recombinase (Supplementary Figure1). In *Gdf9-Cre* mice, Cre was expressed from primordial to later follicular stages. Histological analysis of *Ck2β*^*fl/fl*^*;GCre*^*+*^ mouse ovaries showed loss of CK2β localization in nuclei of oocytes, indicating functional deletion of CK2β (Fig. 1c).

**Fig. 2:**
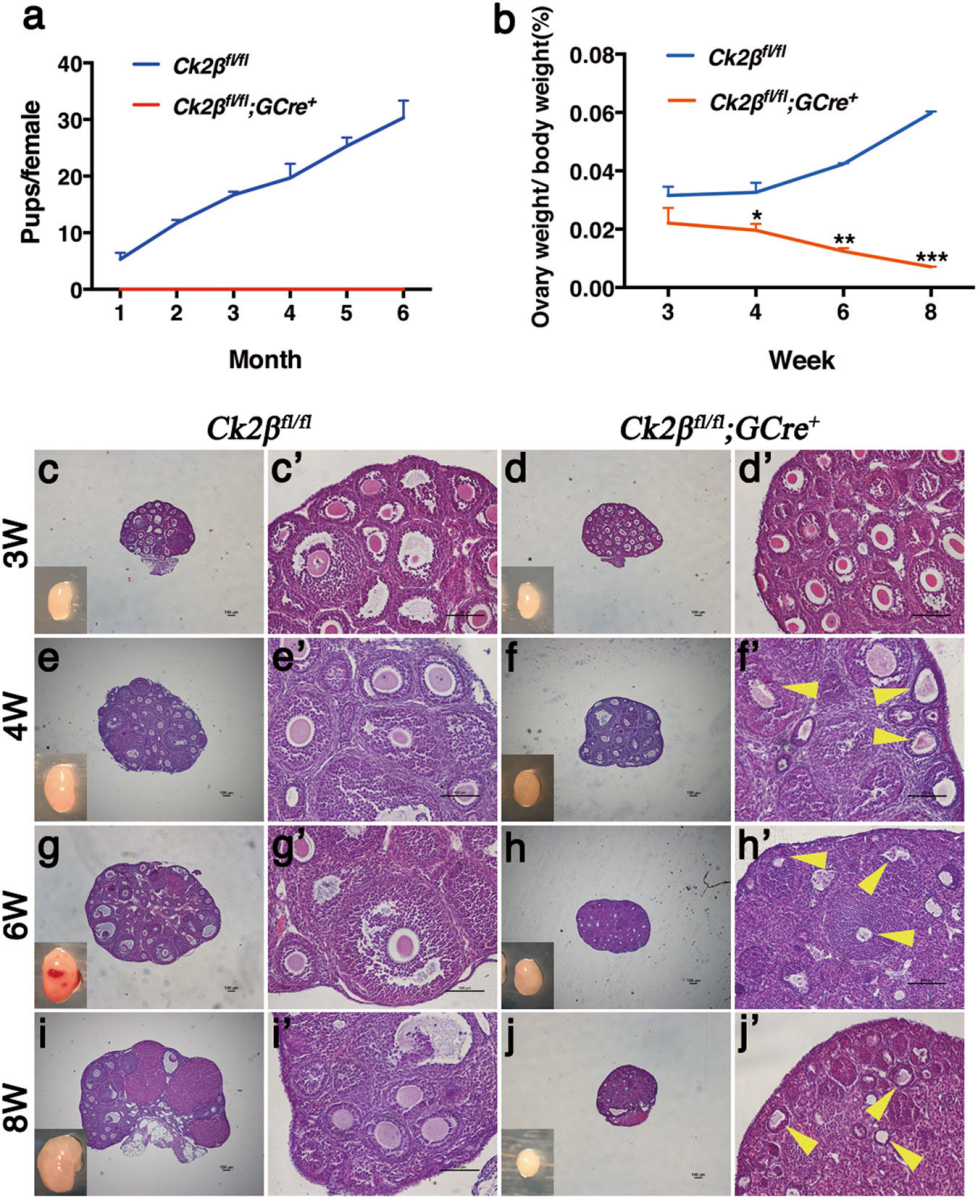
Infertility and follicle atresia in *Ck2β*^*fl/fl*^*;GCre*^*+*^ mice. **a** Comparison of the accumulative number of pups per *Ck2β*^*fl/fl*^ female and *Ck2β*^*fl/fl*^*;GCre*^*+*^ female for 6 months. At least five mice of each genotype were used in this assay. **b** Ovary weight to body weight ratio of *Ck2β*^*fl/fl*^ and *Ck2β*^*fl/fl*^*;GCre*^*+*^ mice at 3, 4, 6, and 8 weeks of age after birth. For each time point, at least three mice of each genotype were used for analysis. Data are presented as the mean ± SEM. *P* < 0.05(*), 0.01(**) or 0.001(***). **c**–**j** Representative ovarian histology of *Ck2β*^*fl/fl*^ and *Ck2β*^*fl/fl*^*;GCre*^*+*^ mice of 3, 4, 6, and 8 weeks of age, respectively. Images **c’**–**j’** correspond to the partial magnification of images **c**–**j**. Yellow arrowheads in **f’**, **h’**, and **j’** indicate atretic follicles. For each time point, at least three mice of each genotype were used for analysis. Scale bar: 100 μm

To observe the effect of oocyte-specific deletion of CK2β on fertility, a breeding assay was conducted by mating *Ck2β*^*fl/fl*^ or *Ck2β*^*fl/fl*^*;GCre*^*+*^ female mice with wild-type males of tested fertility for 6 months. Continuous breeding assessment indicated that *Ck2β*^*fl/fl*^*;GCre*^*+*^ females were completely infertile (Fig. 2a).

### Ablation of *Ck2β* gene expression in oocytes at the primordial follicle stage results in follicle atresia and POF

To determine whether the infertility was due to ovarian dysfunction and consequential functional oocyte loss, we first measured the size of ovaries from *Ck2β*^*fl/fl*^ and *Ck2β*^*fl/fl*^*;GCre*^*+*^ mice. As shown in Fig. 2b, the size of the ovaries of *Ck2β*^*fl/fl*^ mice continued to increase from week 3 to week 8, with a mean ovarian weight ratio of 0.0316%, 0.0326%, 0.0424%, and 0.0598% corresponding to week 3, 4, 6, and 8, respectively. Compared to the control, the ovary size of *Ck2β*^*fl/fl*^*;GCre*^*+*^ mice decreased slightly in week 3 and decreased sharply from week 4 to week 8, with mean ovarian weight ratio of only 0.0221%, 0.0196%, 0.0124%, and 0.0071%. These data revealed that CK2β deletion resulted in atrophy of ovaries in mice.

To clarify the cause of ovarian atrophy, we next examined the morphology of ovaries from both *Ck2β*^*fl/fl*^ and *Ck2β*^*fl/fl*^*;GCre*^*+*^ mice. At 3 weeks of age, histological assessment revealed that *Ck2β*^*fl/fl*^ mice showed normal ovarian morphology characterized by the presence of primordial and activated follicles including primary, secondary, and antral follicles (Fig. 2c, c’). All of these structures were also found in the *Ck2β*^*fl/fl*^*;GCre*^*+*^ mouse ovaries. Although a few follicles underwent atresia, the ovaries looked healthy on the whole (Fig. 2d, d’). At 4 weeks of age, all types of follicles could be found in both *Ck2β*^*fl/fl*^ mice (Fig. 2e, e’) and *Ck2β*^*fl/fl*^*;GCre*^*+*^ mice (Fig. 2f, f’). However, most follicles in the ovaries of *Ck2β*^*fl/fl*^*;GCre*^*+*^ mice showed signs of atresia (Fig. 2f’, yellow arrows) in contrast to control ovaries that contained substantial healthy-looking follicles (Fig. 2e’). At 6 weeks, the time of sexual maturity, massive atretic follicles (Fig. 2h’, yellow arrows) appeared in the ovaries of *Ck2β*^*fl/fl*^*;GCre*^*+*^ mice (Fig. 2h, h’) compared to *Ck2β*^*fl/fl*^ mice (Fig. 2g, g’). Although most atretic follicles maintained follicular structures, the oocytes in atretic follicles were eliminated and the space was filled with granulosa cells (Fig. 2h, h’). By 8 weeks of age, in contrast to *Ck2β*^*fl/fl*^ ovaries (Fig. 2I, i’), almost all types of follicles had been depleted in *Ck2β*^*fl/fl*^*;GCre*^*+*^ ovaries, and it was difficult to find follicular structures in the ovaries of *Ck2β*^*fl/fl*^*;GCre*^*+*^ mice (Fig. 2j, j’).

**Fig. 3:**
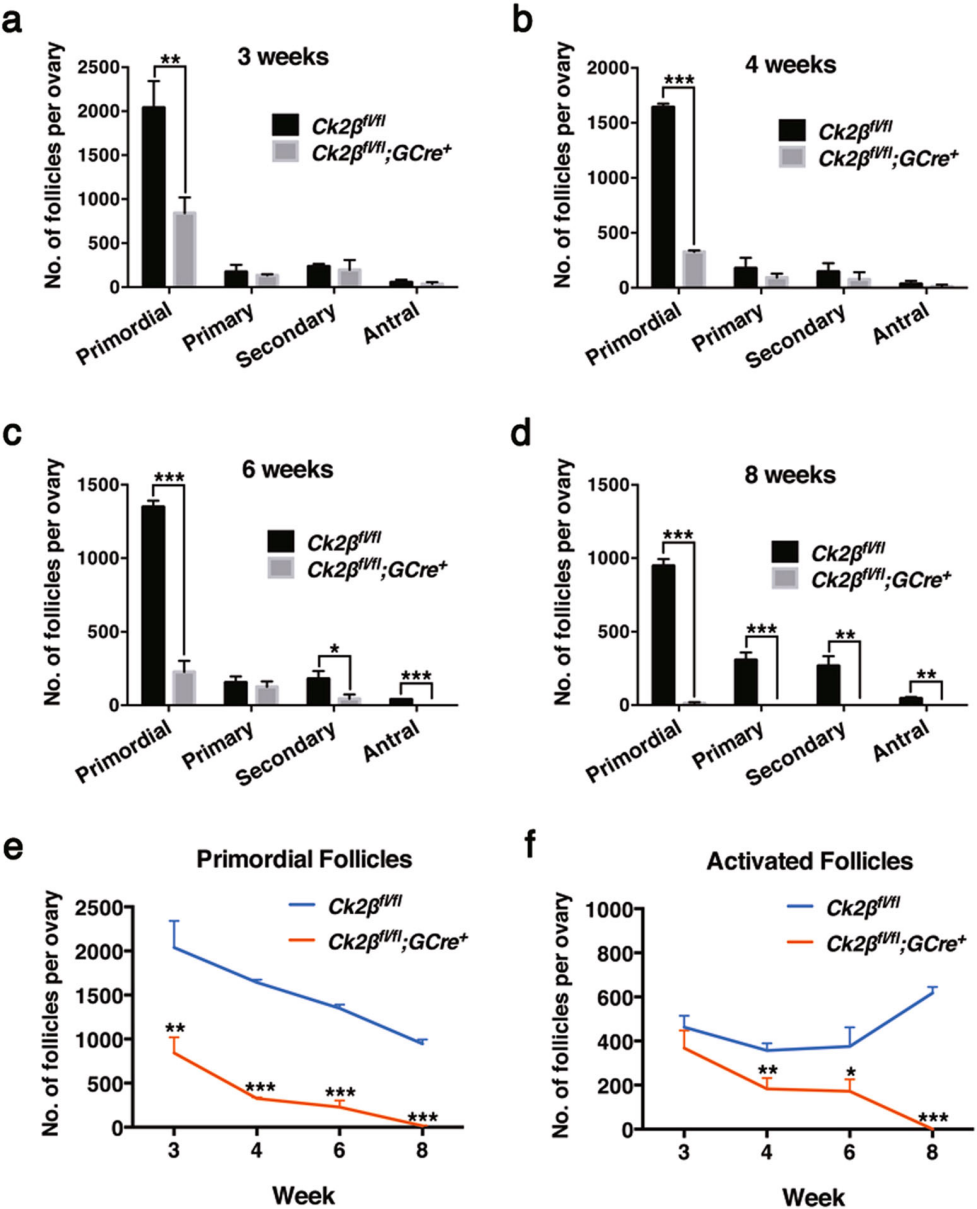
Decreased number of primordial and activated follicles in *Ck2β*^*fl/fl*^*;GCre*^*+*^ mice. **a**–**d** Quantification of numbers of different types of follicles in ovaries at 3 weeks, 4 weeks, 6 weeks, and 8 weeks, respectively. Primordial, primary, secondary, and antral follicles were counted. For each time point, at least three mice of each genotype were used for analysis. Data are presented as the mean ± SEM. *P* < 0.05(*), 0.01(**) or 0.001(***). **e**–**f** Quantification of numbers of primordial follicles (**e**) and activated follicles (**f**) in ovaries at 3 weeks, 4 weeks, 6 weeks, and 8 weeks, respectively. For each time point, at least three mice of each genotype were used for analysis. Data are presented as the mean ± SEM. *P* < 0.05(*), 0.01(**) or 0.001(***)

Quantitative analysis revealed that the numbers of primary, secondary, and antral follicles in the ovaries of *Ck2β*^*fl/fl*^*;GCre*^*+*^ mice were similar to those of *Ck2β*^*fl/fl*^ mice at 3 and 4 weeks, but the number of primordial follicles within the ovaries of *Ck2β*^*fl/fl*^*;GCre*^*+*^ mice was markedly reduced as compared to *Ck2β*^*fl/fl*^ mice (Fig. 3a, b). At 6 weeks, in addition to primordial follicles, significant differences were also observed in the secondary and antral follicles (Fig. 3c). By 8 weeks of age, all types of follicles in ovaries of *Ck2β*^*fl/fl*^*;GCre*^*+*^ mice were significantly decreased (Fig. 3d). In general, the primordial follicles reduction occurred at 3 weeks of age and continued decreasing until depletion of the primordial follicle pool at young adulthood (8 weeks) in *Ck2β*^*fl/fl*^*;GCre*^*+*^ mice compared to *Ck2β*^*fl/fl*^ mice (Fig. 3e). The activated follicle reduction appeared at the time of onset of sexual maturity (4 weeks) and displayed a similar decreased trend from week 4 to week 6 for primordial follicles in the ovaries of *Ck2β*^*fl/fl*^*;GCre*^*+*^ mice (Fig. 3f). This phenotype resembles premature ovarian failure (POF) in humans.

**Fig. 4:**
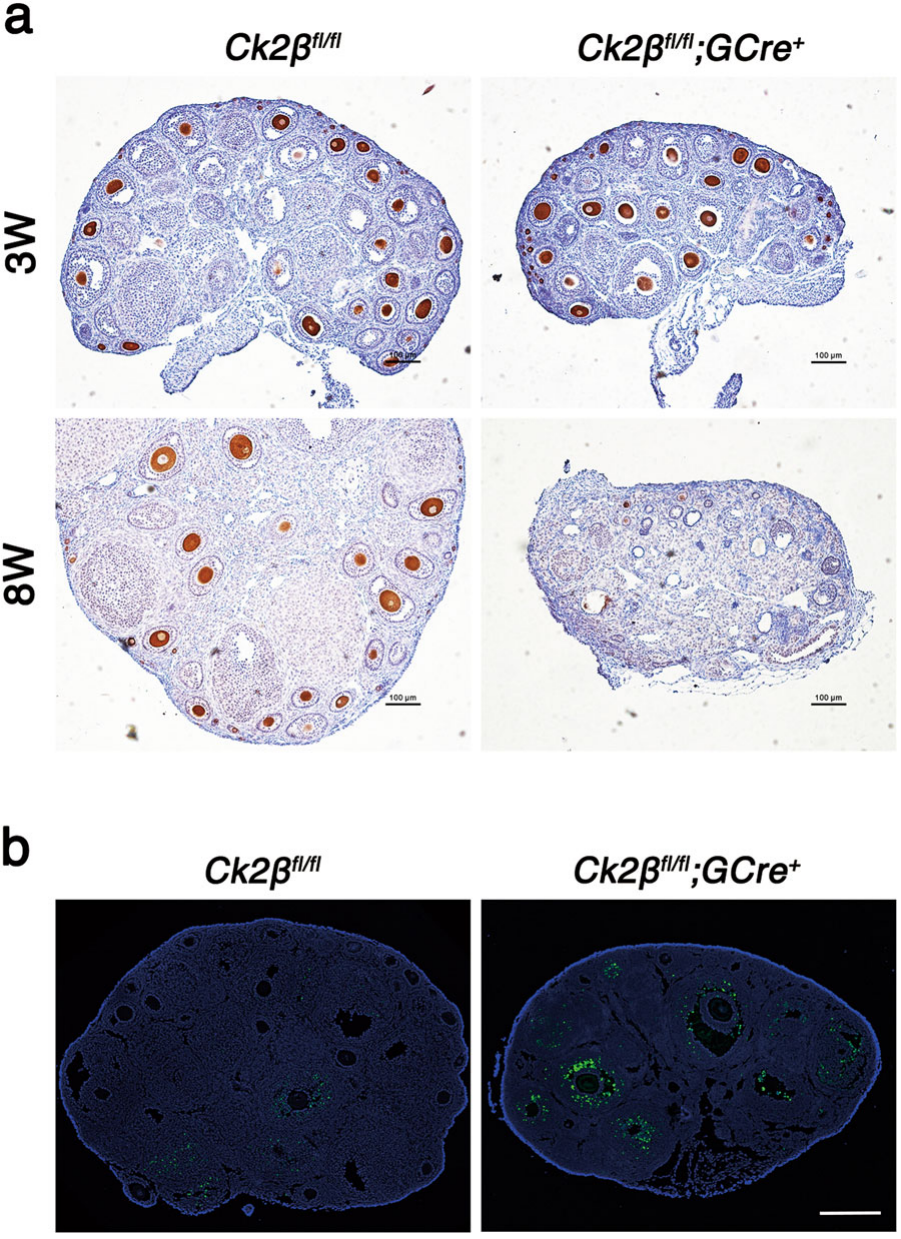
Premature ovarian failure in *Ck2β*^*fl/fl*^*;GCre*+ mice. **a** Germ cells marker MVH immunohistochemistry of ovaries of *Ck2β*^*fl/fl*^ and *Ck2β*^*fl/fl*^*;GCre*^*+*^ at 3 and 8 weeks after birth. At least three mice of each genotype were used in this assay. Scale bar: 100 μm. **b** TUNEL immunofluorescence staining of the ovaries of *Ck2β*^*fl/fl*^ and *Ck2β*^*fl/fl*^*;GCre*^*+*^ at 4 weeks after birth. Green: TUNEL-positive signal; Blue: DAPI. At least three mice of each genotype were used for analysis. Scale bar: 100 μm

The histological analysis indicated that absence of CK2β in oocytes caused follicular atresia and POF. To confirm these observations, we performed immunohistochemistry of the germ cell marker MVH on 3- and 8-week-old ovarian sections. As shown in Fig. 4a, in ovaries of *Ck2β*^*fl/fl*^*;GCre*^*+*^ mice at 3 weeks of age, most follicles including primordial, primary, secondary, and antral follicles showed MVH-positive staining, suggesting that these follicles were healthy. However, at 8 weeks of age, there were few MVH-positive primordial or primary follicles scattering in the cortical region in ovaries of *Ck2β*^*fl/fl*^*;GCre*^*+*^ mice, which was symptomatic of POF. TUNEL assay on ovarian sections showed that increased granulosa cell apoptosis occurred in ovaries of *Ck2β*^*fl/fl*^*;GCre*^*+*^ mice at 4 weeks of age compared to ovaries in *Ck2β*^*fl/fl*^ mice, which was caused by growing follicle atresia (Fig. 4b). The above data demonstrate that the accelerated demise of primordial follicles and defective survival of growing follicles may be responsible for infertility of *Ck2β*^*fl/fl*^*;GCre*^*+*^ female mice.

### *Ck2β* deletion causes PI3K/AKT signaling hypoactivation

**Fig. 5:**
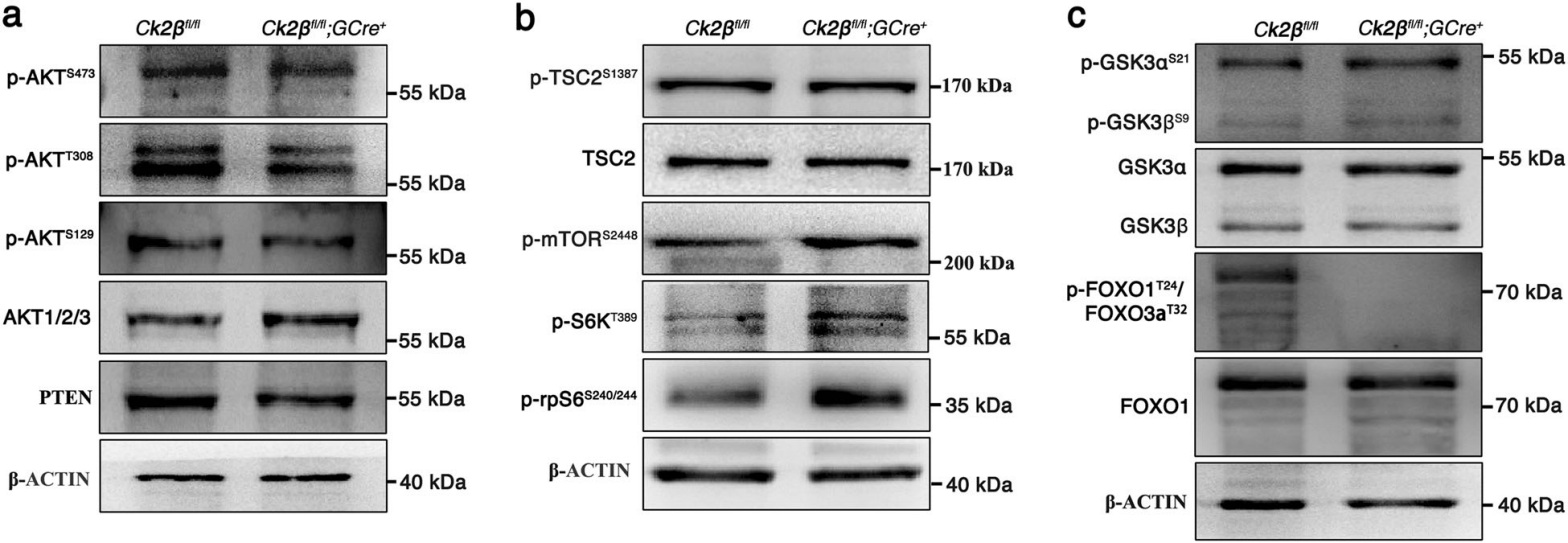
Downregulation of PI3K/AKT signaling in ovaries from *Ck2β*^*fl/fl*^*;GCre*^*+*^ mice. Immunoblotting detection of PI3K/AKT signaling in ovaries of *Ck2β*^*fl/fl*^ and *Ck2β*^*fl/fl*^*;GCre*^*+*^ at 2 weeks after birth. The ovary lysates were collected at least from three mice of each genotype and immunoblotted for p-AKT (S473), p-AKT (T308), p-AKT (S129), AKT1/2/3, PTEN, p-TSC2 (S1387), TSC2, p-mTOR (S2448), p-S6K (T389), p-rpS6 (S240/244), p-GSKα/β (S21/9), GSKα/β, p-FOXO1 (T24)/FOXO3a (T32), FOXO1, and β-actin. Levels of β-action were used as internal control. Each experiment was repeated at least 2–3 times. Molecular mass is given in kilo Daltons

According to the above-mentioned research, *Ck2β*^*fl/fl*^*;GCre*^*+*^ mice displayed defects in primordial follicle survival. Accumulating literature indicates that PI3K/AKT signaling plays a vital role in regulating the survival of primordial follicles during their long dormancy^54–56^. In view of the involvement of CK2 in the PI3K/AKT pathway at a cellular level, we first focus our attention on the PI3K/AKT pathway. Accordingly, we performed immunoblotting analysis using ovaries from *Ck2β*^*fl/fl*^ and *Ck2β*^*fl/fl*^*;GCre*^*+*^ mice at 2 weeks of age. It showed that the levels of phosphorylated AKT (S473 and S129) decreased slightly, whereas phosphorylated AKT (T308) was markedly reduced (Fig. 5a); when considering the elevated total level of AKT1/2/3 protein (Fig. 5a), the relative level of phosphorylated AKT (S473, T308, and S129) was decreased more obviously in CK2β mutant ovaries. PI3K/AKT regulator PTEN was also detected and it showed decreased expression (Fig. 5a). Subsequently, the downstream of PI3K/AKT pathway was detected. We first examined the TSC2/mTOR signaling which was critical to oocyte survival. The results showed that the activity of mTOR/rpS6 signaling pathway was enhanced in mutant ovaries, as indicated by elevated levels of phosphorylated mTOR (S2448), phosphorylated S6K (T389), and phosphorylated rpS6 (Ser240/244) (Fig. 5b). However, the levels of phosphorylated TSC2 (S1387) displayed no difference between mutant and control ovaries (Fig. 5b). These results were inconsistent when considering that mTOR was negatively regulated by TSC2. The upregulated mTOR/S6K/rpSK signaling may result from feedback effects on defective follicle survival. Afterward, other AKT substrates contributing to cell survival were further detected. As the first reported AKT substrate which is important to cell survival, two isoforms of glycogen synthase kinase 3 (GSK3), GSK3α and GSK3β, were detected. The results showed that the level of GSK3α expressed in ovaries was higher than GSK3β (Fig. 5c), and the expression levels of both phosphorylated GSK3α/β (S21/9) and total GSK3α/β protein displayed no variation in control and mutant mice (Fig. 5c). We next detected FOXO proteins which are also important cell survival-related AKT substrates. The examination of the levels of phosphorylated FOXO1 (T24)/FOXO3a (T32) found that they were significantly decreased (Fig. 5c), while the levels of total FOXO1 protein did not show obvious changes (Fig. 5c).

### *Ck2β* depletion results in accumulated DNA damage in oocytes

**Fig. 6:**
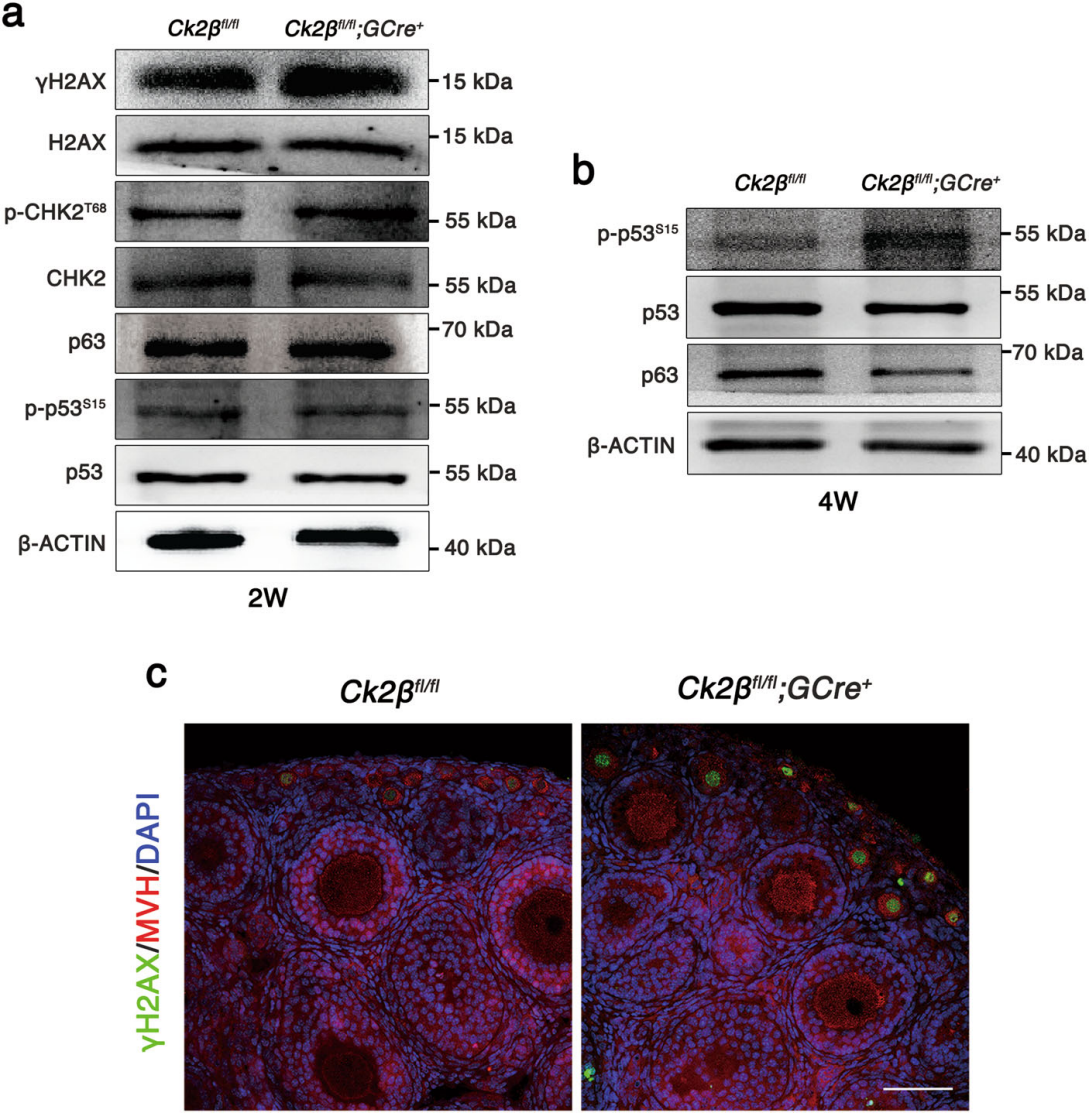
Impaired follicle survival in *Ck2β*^*fl/fl*^*;GCre*^*+*^ mice involved in DNA damage response. **a** Immunoblotting analysis of DNA damage response signaling in ovaries of *Ck2β*^*fl/fl*^ and *Ck2β*^*fl/fl*^*;GCre*^*+*^ mice at 2 weeks after birth. The ovary lysates were collected at least from three mice of each genotype and immunoblotted for γH2AX, H2AX, p-CHK2 (T68), CHK2, p63, p-p53 (S15), p53 and β-actin. Level of β-actin was detected as internal control. Each experiment was repeated at least 2–3 times. Molecular mass is given in kilo Daltons. **b** Immunoblotting analysis of the expression of p53 and p63 in ovaries of *Ck2β*^*fl/fl*^ and *Ck2β*^*fl/fl*^*;GCre*^*+*^ at 4 weeks after birth. The ovary lysates were obtained from at least three mice of each genotype and immunoblotted for p63, p-p53 (S15), p53 and β-actin. Level of β-actin was used as internal control. Each experiment was repeated at least three times. Molecular mass is given in kilo Daltons. **c** MVH and γH2AX immunofluorescent staining of 2-week-old ovarian sections from *Ck2β*^*fl/fl*^ and *Ck2β*^*fl/fl*^*;GCre*^*+*^ mice. Green: γH2AX; Red: MVH; Blue: DAPI. At least three mice of each genotype were used in this assay. Scale bar: 50 μm

Double-strand breaks derived from unrepaired meiotic or environmental stress could result in oocyte elimination and female infertility through CHK2-dependent activation of p53 or p63^57^. Considering that CK2 is involved in the DNA damage response pathway^32,34–36^, we wonder whether *Ck2β*^*−/−*^ oocyte depletion relates to this pathway. Thus, we first performed immunoblotting using ovary lysates from *Ck2β*^*fl/fl*^ and *Ck2β*^*fl/fl*^*;GCre*^*+*^ mice at 2 weeks of age. As shown in Fig. 6a, the levels of phosphorylated CHK2 (T68) were slightly increased and γH2AX was significantly upregulated in ovaries of *Ck2β*^*fl/fl*^*;GCre*^*+*^ mice, whereas the levels of p63 and phosphorylated p53 (S15) showed no difference in ovaries of *Ck2β*^*fl/fl*^ and *Ck2β*^*fl/fl*^*;GCre*^*+*^ mice. However, by 4 weeks of age, the level of p63 was downregulated while phosphorylated p53 (S15) was upregulated in ovaries of *Ck2β*^*fl/fl*^*;GCre*^*+*^ mice (Fig. 6b), consistent with the antagonizing relationship between p53 and p63^57^. Moreover, we confirmed our findings using immunofluorescence analysis. As indicated in Fig. 6c, the γH2AX signals in small oocytes from *Ck2β*^*fl/fl*^*;GCre*^*+*^ mice at 2 weeks of age were remarkably enhanced. Taken together, these data suggest that CK2β depletion causes DSBs accumulation and failed activation of the DNA damage response pathway.

### Oocyte-specific deletion of CK2β causes a striking reduction in CK2α but not CK2α’ expression

**Fig. 7:**
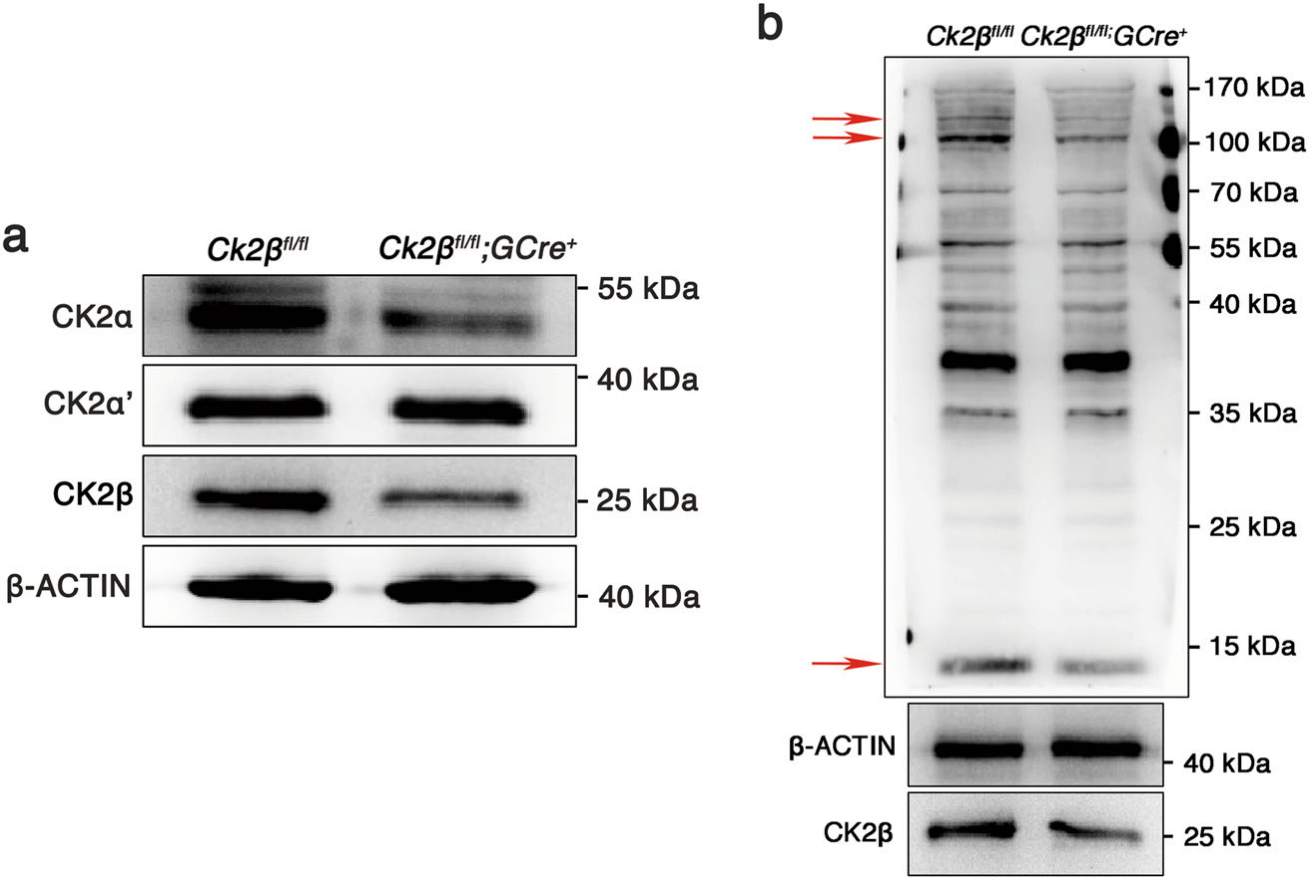
Detection of expression of CK2 subunits and CK2 activity in *Ck2β*^*fl/fl*^*;GCre*^*+*^ mouse ovaries. **a** Immunoblotting detection of the expression of CK2α and CK2α’ in ovaries of *Ck2β*^*fl/fl*^ and *Ck2β*^*fl/fl*^*;GCre*^*+*^ at 2 weeks after birth. **b** Immunoblotting detection of CK2 activity in ovaries of *Ck2β*^*fl/fl*^ and *Ck2β*^*fl/fl*^*;GCre*^*+*^ at 2 weeks after birth. The obviously altered bands were marked with red arrows. The ovary lysates were obtained from at least three mice of each genotype and immunoblotted for CK2α, CK2α’, CK2β, and phospho-CK2 substrate, β-actin. Level of β-actin was used as internal control. Each experiment was repeated at least three times. Molecular mass is given in kilo Daltons

To explore the forms of CK2 functions in the mouse ovary, immunoblotting was carried out to detect protein levels of CK2α, CK2α', and CK2β in ovaries of *Ck2β*^*fl/fl*^ and *Ck2β*^*fl/fl*^*;GCre*^*+*^ mice (Fig. 7). As expected, the level of CK2β protein was dramatically reduced in ovary extracts prepared from *Ck2β*^*fl/fl*^*;GCre*^*+*^ mice compared with *Ck2β*^*fl/fl*^ mice. The low level of CK2β protein that was detected in the ovary extracts collected from *Ck2β*^*fl/fl*^*;GCre*^*+*^ mice likely came from granulosa cells in which CK2β was not deleted. Meanwhile, the level of CK2α protein was significantly downregulated in ovaries of *Ck2β*^*fl/fl*^*;GCre*^*+*^ mice, while the levels of CK2α' protein in ovaries of *Ck2β*^*fl/fl*^*;GCre*^*+*^ mice showed no variation compared with *Ck2β*^*fl/fl*^ mice. Immunoblotting analysis of phospho-CK2 substrate using ovaries from control and mutant mice found that CK2 activity was largely reduced (Fig. 7b). These data suggest that CK2 is presumably functioning in oocytes in the forms of α_2_β_2_ and the reduced expression of CK2α protein in ovaries of *Ck2β*^*fl/fl*^*;GCre*^*+*^ mice is probably due to degradation resulting from decreased stability of CK2α protein without CK2β.

## Discussion

In humans, the primordial germ cells (PGCs) migrate to gonadal ridges and are enclosed by pregranulosa cells to form primordial follicles. Most ovarian primordial follicles are maintained in a quiescent state, providing as a reserve for a woman’s reproductive life^58^. Premature depletion of the ovarian reserve incurs cessation of ovarian function, resulting in POF^59^. Elucidating the mechanisms that control the dormancy and survival of primordial follicles is critical for understanding of ovarian biology. In this study, using *Gdf9* promotor-driven Cre recombinase, we successfully deleted CK2β in oocytes from the primordial follicle stage, which facilitated investigation on the roles of CK2β in folliculogenesis. We found that CK2β mutant females showed defective follicular survival and sterility.

Morphological observation revealed that the ovary size of CK2β mutant mice started to decrease from 3 weeks after birth and eventually became reduced to about 1/4 of that in control mice at 8 weeks, which was consistent with histological observations and follicle counts. At 3 weeks, most follicles looked healthy but the primordial follicles in ovaries of CK2β mutant mice were markedly reduced compared to control mice. Starting at 4 weeks, the ovaries of CK2β mutant mice showed increasing numbers of atretic follicles, which were eliminated quickly, resulting in the decrease of the number of activated follicles.

Previous researches reveal that PI3K signaling is critical to control the survival of primordial follicles, since suppressed or elevated PI3K/AKT signaling leads to premature depletion of follicles, causing POF^54–56^. In this study, we systematically analyze the PI3K/AKT signaling. Immunoblotting results showed that *Ck2β* depletion caused downregulation of phosphorylated AKT (S473, T308, and S129). Previous studies find that CK2 can phosphorylate AKT/PKB at Ser129 and such phosphorylation of AKT prevents AKT Thr308 dephosphorylation and enhances the catalytic activity of AKT/PKB^24,30,37^. Previous studies also show that CK2 can phosphorylate PTEN at several sites and such phosphorylation prevents PTEN degradation and inhibits its activity^38–40^. Consequently, deletion of CK2β causes low stability and high activity of PTEN, leading to reduced level of PTEN protein and downregulated AKT phosphorylation.

Studies of targets of AKT found that the activity of TSC2 was not changed but its downstream pathway mTOR/S6K/rpSK signaling was significantly enhanced in CK2β mutant ovaries. These findings are in conflict with previous reports which suggest that elevated mTOR/S6K/rpSK signaling is responsible for oocyte growth and follicular activation and suppressed mTOR/S6K/rpSK signaling leads to loss of primordial follicles^54,55^. The upregulated mTOR/S6K/rpSK signaling may result from feedback effects to defective follicle survival. Further studies of other targets of AKT found that the levels of phosphorylated GSK3α/β (S21/9) were not changed. However, phosphorylated FOXO1 (T24)/FOXO3a (T32) were downregulated in CK2β mutant mice. CK2β deletion downregulates activated AKT, which in turn inhibits FOXO phosphorylation and keeps FOXO1 staying in nucleus^60,61^. Unphosphorylated FOXO proteins trigger expression of genes that are crucial for the induction of apoptosis, such as FASL and BIM^62^. From above results, CK2β regulates follicular survival at least partly via PI3K/AKT/FOXO pathway, although details remain to be elucidated.

The oocytes derived from primordial follicle are arrested at the diplotene stage of prophase of first meiotic division for months, even years, after birth depending on species. The oocytes at the diplotene stage have finished synapsis that relies on homologous recombination, a high-fidelity DNA double-strand breaks (DSBs) repair process. Any homolog synapsis or DSB repair errors would prompt DNA damage checkpoints to eliminate defective meiotic oocytes, which is mediated by the CHK2-p53/p63 pathway^57,63^. Considering the involvement of CK2 in the DNA damage response pathway in other species^32,34–36^, we then studied this pathway in CK2β mutant ovaries. In our study, oocyte-specific deletion of CK2β from the primordial follicle stage caused elevated γH2AX and phosphorylated CHK2 (T68) signals in ovaries and elevated γH2AX signals in small oocytes from mice at 2 weeks of age, indicating accumulated DSBs. By 4 weeks of age, the DNA damage response pathway was significantly elevated, indicated by the upregulated level of phosphorylated p53 (S15) in ovaries of *Ck2β*^*fl/fl*^*;GCre*^*+*^ mice. Accordingly, the follicular atresia at least early follicle atresia may be partly dependent on DNA damage response pathway.

In this study, as expected, the level of CK2β protein was significantly reduced in CK2β mutant ovaries compared to controls. However, the level of CK2α also showed an obvious decrease despite of no difference in the levels of CK2α’ in CK2β mutant ovaries and control ovaries. This is consistent with a previous report in which CK2α’^−/−^ females show normal fertility^51^. The above data reveal that CK2 presumably functions in oocytes in the forms of α_2_β_2_, but since CK2α^−^^/−^ embryos die at the embryonic stage^50^, oocyte-specific knockdown of CK2α is necessary to validate the hypothesis.

In summary, we identified CK2β as a key protein safeguarding mouse follicle survival and fertility. Our data provide new insights into occurrence, diagnosis, and treatment of POF.

## Materials and methods

### Mice

Mice lacking *Ck2β* in oocytes (referred to as *Ck2β*^*fl/fl*^*;GCre*^*+*^) were obtained by crossing previously reported *Ck2β*^*fl/fl*^ mice^53^ with *Gdf9*-*Cre* (C57BL6 background) mice^64^. The *Ck2β*^*fl/fl*^ female mice were used as control group. DNA extraction from mouse tail was used to genotype *Ck2β*^*∆*^, *Ck2β*^*fl*^, and Gdf9-Cre alleles, respectively. The primer pair for *Ck2β*^*∆*^ allele was forward: 5′-GAGGGCATAGTAGATATGAATCTG-3′ and reverse: 5′-GGATAGCAAACTCTCTGAG-3′. The primer pair for *Ck2β*^*fl*^ allele was forward: 5′-ATGAGTAGCTCTGAGGAGGTG-3′ and reverse: 5′-GGATAGCAAACTCTCTGAG-3′. The primer pair for *Gdf9-Cre* allele was forward: AGGCATGCTTGAGGTCTGAT, and reverse: CACAGTCAGCAGGTTGGAGA. All animal operations were approved by the Animal Research Committee principles of the Institute of Zoology, Chinese Academy of Sciences.

### Antibodies

Rabbit monoclonal anti-CK2β antibody (AJ1128b; ABGENT); mouse monoclonal anti-β-actin antibody (sc-47778; Santa Cruz); mouse monoclonal anti-MVH antibody (ab27591; abcam); rabbit monoclonal anti-p-Akt (S473) antibody (4046; Cell Signaling Technology, Inc.); rabbit monoclonal anti-p-Akt (T308) (13038; Cell Signaling Technology, Inc.); rabbit monoclonal anti-p-Akt (S129) (ab133458; abcam); rabbit monoclonal anti-AKT1/2/3 antibody (ab32505; abcam); rabbit monoclonal anti-PTEN antibody (9559; Cell Signaling Technology, Inc.); rabbit polyclonal anti-p-TSC2 (S1387) antibody (5584; Cell Signaling Technology, Inc.); rabbit monoclonal anti-TSC2 antibody (4308; Cell Signaling Technology, Inc.); rabbit monoclonal anti-p-mTOR (S2448) antibody (5536; Cell Signaling Technology, Inc.); rabbit monoclonal anti-p-S6K (T389) antibody (9234; Cell Signaling Technology, Inc.); rabbit monoclonal anti-p-rpS6 (S240/244) antibody (5364; Cell Signaling Technology, Inc.); rabbit polyclonal anti-GSK3α/β (S21/9) antibody (9331; Cell Signaling Technology, Inc.); rabbit monoclonal anti-GSK3α/β antibody (5676; Cell Signaling Technology, Inc.); rabbit polyclonal anti-p-FOXO1 (T24)/FOXO3a (T32) antibody (9464; Cell Signaling Technology, Inc.); rabbit monoclonal anti-FOXO1 antibody (2880; Cell Signaling Technology, Inc.); rabbit monoclonal anti-γH2AX antibody (9718; Cell Signaling Technology, Inc.); rabbit monoclonal anti-H2AX antibody (7631; Cell Signaling Technology, Inc.); rabbit polyclonal anti-p-CHK2 (T68) antibody (BS4043; Bioworld Technology, Inc.); rabbit polyclonal anti-CHK2 antibody (BS6791; Bioworld Technology, Inc.); rabbit monoclonal anti-p-p53 (S15) (12571; Cell Signaling Technology, Inc.); rabbit polyclonal anti-p53 antibody (BS3156; Bioworld Technology, Inc.); rabbit monoclonal anti-p63 (ab124762; abcam); mouse monoclonal anti-CK2α antibody (ab70774; abcam); rabbit polyclonal anti-CK2α’ antibody (BS6571; Bioworld Technology, Inc.); rabbit monoclonal anti-phospho-CK2 substrate [(pS/pT)DXE] (8738; Cell Signaling Technology, Inc.); secondary antibodies were purchased from Zhongshan Golden Bridge Biotechnology Co, Ltd (Beijing).

### Immunoblotting

Ovary extracts were prepared using a homogenizer in RIPA buffer supplemented with protease and phosphatase inhibitor cocktail (Roche Diagnostics). After transient ultrasound, the ovary lysates were incubated on ice for 30 min and then centrifuged at 4 °C, 12,000 rpm for 20 min. The supernatant was transferred to a new tube and equal volume loading buffer was added. After being boiled at 95 °C for 10 min, the protein lysates were used for immunoblotting analysis. Immunoblotting was performed as described previously^65^. Briefly, the separated proteins in SDS-PAGE were electrically transferred to a polyvinylidene fluoride membrane. After incubation with primary and secondary antibodies, the membranes were scanned with Bio-Rad ChemiDoc XRS+.

### Hematoxylin and eosin staining and quantification of ovarian follicles

Ovaries were dissected from *Ck2β*^*fl/fl*^ and *Ck2β*^*fl/fl*^*;GCre*^*+*^ mice immediately after killing. The ovaries were fixed in 4% formaldehyde overnight at 4 °C, dehydrated in an ethanol series and embedded in paraffin. Paraffin-embedded ovaries were cut into sections of 8-μm thickness and mounted on glass slides. After 48 °C overnight drying, the sections were deparaffinized in xylene, hydrated by a graded alcohol series and stained with hematoxylin and eosin for histological analyses. Ovarian primordial, primary, secondary, and antral follicles were counted in every fifth section of an ovary. Quantification of ovarian follicles was performed as previously reported^66,67^. In each section, follicles that contained oocytes with clearly visible nuclei were scored and the cumulative number of follicles were multiplied by a correction factor of 5 to represent the estimated number of total follicles in an ovary.

### Immunohistochemistry and immunofluorescence

Ovaries used for immunostaining were fixed in 4% paraformaldehyde (pH 7.4) overnight at 4 ℃, dehydrated, and embedded in paraffin. Paraffin-embedded ovaries were cut into sections of 5-μm thickness. Then, the sections were deparaffinized, immersed in sodium citrate buffer (pH 6.0), and heated for 15 min in a microwave for antigen retrieval. After blocking with 5% donkey serum albumin, sections were incubated with primary antibodies at 4 °C overnight. For immunohistochemistry, the sections were treated with 3% H_2_O_2_ to eliminate internal peroxidase activity and incubated with an appropriate horseradish peroxidase (HRP)-conjugated secondary antibody. Finally, the signal of primary antibody was detected by the Vectastain ABC kit (Vector Laboratories, CA, USA) and the sections were counterstained with hematoxylin. Images were captured using a Nikon DS-Ri1 CCD camera. For immunofluorescence, the sections were incubated with an appropriate FITC-conjugated secondary antibody. The nuclei were stained with DAPI. Images were captured using a laser scanning confocal microscope (Zeiss 780 META).

### TUNEL assay

TUNEL assay was carried out in accordance to the DeadEnd^TM^ Fluorometric TUNEL System (Promega BioSciences, Madison, WI, USA). Images were captured using a laser scanning confocal microscope (Zeiss 780 META).

### Breeding assay

In the breeding assay, 6–8 week-old *Ck2β*^*fl/fl*^ and *Ck2β*^*fl/fl*^*;GCre*^*+*^ female mice were mated to 8-week-old C57BL/6J wild-type male mice with known fertility. At least five mice of each genotype were used in this assay. For 6 months, the cages were monitored daily for recording the number of pups and litter size.

### Statistical analysis

All experiments were performed at least three times. Paired two-tailed Student’s *t* test was used for statistical analysis. Data were presented as mean ± SEM and *P* < 0.05(*), 0.01(**), or 0.001(***) was considered statistically significant.

## Electronic supplementary material

Supplemental Figure S1
