# Supplementary figures and images for: Ablation of beta subunit of protein kinase CK2 in mouse oocytes causes follicle atresia and premature ovarian failure
Source: Cell Death Dis. 2018 May 3;9(5):508. doi: 10.1038/s41419-018-0505-1 (PMC5938699; doi:10.1038/s41419-018-0505-1)

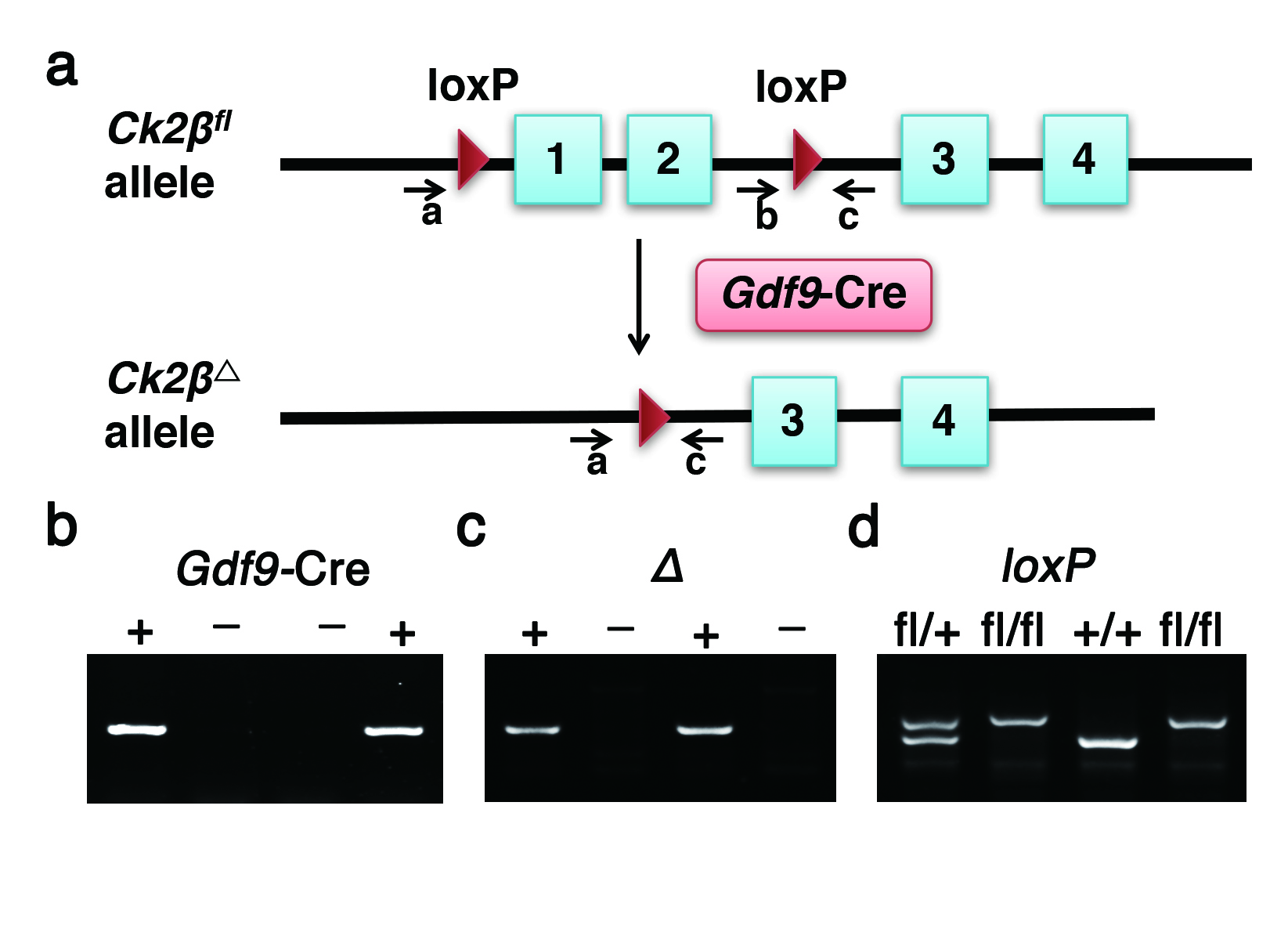

Supplement: Supplementary file 1 — Supplemental Figure S1 [file 41419_2018_505_MOESM1_ESM.jpg]
